# Renal Calculus

**Published:** 1890-04-05

**Authors:** 


### RENAL CALCULUS.

The second part of a paper by Mr. Jacobson, assistant-surgeon to Guy's Hospital, on "Nephrolithotomy," is devoted to "the chief conditions stimulating renal calculas." These are: *First, lithiasis*, and to a less degree, *oxaluria*. The passage of uric acid crystals may produce hæmaturia, also lumbar and testicular pains, which may give rise to a suspicion of calculus. Mr. Jacobson says "The diagnosis will not be difficult by watching the results of treatment, which only gives relief in the one but clears up the other." Of this, however, we are by no means certain, for treatment does not always clear away lithic acid from the system. "Exercise, again, is a test. A patient with renal calculus who declines or is unsuited for operative treatment is often much crippled in carrying out palliative treatment, and made worse by the exercise which is otherwise so essential to him. *Secondly, tubercular kidney.* Lumbar pain, and tenderness, frequent micturition, hæmaturia, are all common to tubercular kidney and to renal calculus. Mr. Jacobson says the chief aids to diagnosis are "(a) the pyuria, (b) careful examination of the urine, (c) early pyrexia, (d) early exploration of the kidney." In tubercular kidney, pyuria is usually present early in the case with albumen, but not much hæmaturia; on examination of urine debris of connective tissue or *bacillus tuberculosis* may perhaps be detected. The pyrexia of tubercular kidney is intermittent. *Thirdly, pyelitis not tubercular.* The symptoms often simulate those of renal calculus. But pyelitis may follow a gonorrhœa, perhaps a previous stone, or occur to women in pregnancy. *Fourthly, movable kidney*, especially if associated with neuralgia, pyelitis, or, if occurring with some of the reflex causes, of nephralgia. Mr. Jacobson does not say how this is to be diagnosed, but the mobility of the tumour, the occasional disappearance, the unchanging character of the tumour, clear percussion, and flattening of the renal region on one side, are distinctive. In a recent article on movable kidney, by Dr. Drummond, Newcastle-on-Tyne, the author mentions that the usual symptoms are aching, dragging, burning pain, aggravated by exertion. The hypochondrium is most affected, but the pain often travels to the shoulder or to the ovary. Dyspeptic symptoms are often prominent. Sometimes there is intestinal mucus catarrh. Most patients suffering from movable kidney are nervous, "and might very properly be described as decided neurotics." In three out of thirty-one cases hæmaturia was observed, and in another instance Bright's disease. Although Dr. Drummond's paper is a lengthy one, and the details of thirty-one cases are given, Dr. Drummond does not say anything regarding the difficulty of diagnosing this malady from calculus of the kidney. Neither do the details of the cases show there should be any such difficulty. As a matter of experience this does not very frequently occur. *Fifthly, aching kidney.* The chief points in the diagnosis are the absence of blood and pus, and the fact that the aching often occurs in women, only at menstrual periods. *Sixthly, nephralgias* due to disease in parts adjacent to the kidneys. Such are duodenal ulcers, and intestinal irritation from worms. *Seventhly, gall stones*, retained in the gall bladder. Repeated attacks of intestinal colic, especially with nausea, may be the only symptoms of either a renal or biliary calculus. There are, however, usually some differences which Mr. Jacobson does not point out. The pain from gall stone passes from the right of the pit of the stomach shooting through to the back, whereas pain from kidney calculi or gravel shoots from the loins down the groin and thighs, where there is often numbness not observed in connection with gall stone. In the latter condition there is not frequent desire to make water, in kidney affection there is. There is also usually a previous history of gravel or of gall stone as the case may be. *Eighthly*, the difficulty which may arise in diagnosing spinal caries and renal calculus has not been sufficiently recognised. Where a local patch of caries of a vertebral body exists, and when deep suppuration occurs and presses on the kidneys, nearly all the symptoms of a calculus have been present. Mr. Jacobson does not inform us how to diagnose between the two, but we should imagine the previous history of the patient would be a most important, if not a conclusive evidence. *Ninthly, interstitial shrinking nephritis* may simulate renal calculus, both by hœmaturia and pain. Mr. Bowlby has pointed out the following as distinguishing this condition from calculus: "The specific gravity of the urine, after the blood has cleared up, only 1,008 to 1,015; tortuous arteries, cardiac hypertrophy, and high arterial tension; blurred, ill-defined discs, some retinitis and effusion amongst the blood-vessels." *Tenthly, malignant disease.* Mr. Jacobson sketches a case of the kind where, the symptoms being very much those of renal calculus, malignant disease involving the last dorsal nerve was found after death. Now the surgery of the kidney has recently made great advances in the hands of Mr. Jacobson, and of several others who have applied themselves to this interesting subject; and surgery of the kidney is chiefly connected with calculi. But we may see from the above how difficult the diagnosis of a kidney calculus may be. There is also another condition, not mentioned by the author, which also adds to the difficulty. This is the possibility of calculous degeneration without the formation of any stone to be detected by exploration. Dr. Ralfe has pointed out the solvent action of distilled water, and we cannot help thinking that, as many matters pass out with the urine, we may eventually find a solvent for deposits in the kidneys, which will prevent the recourse to operative procedure.

